# Zona pellucida is required for oocyte actin cortex and oocyte-somatic cell interactions during oocyte growth

**DOI:** 10.1038/s41420-026-03124-9

**Published:** 2026-04-20

**Authors:** Shoujing Liang, Weixi Xiang, Ruping Quan, Jiayu Su, Chao Lv, Hualin Huang, Hongmei Xiao

**Affiliations:** 1 Institute of Reproductive and Stem Cell Engineering, Xiangya School of Basic Medical Sciences, Central South University, Changsha, Hunan China; 2 Department of Reproductive Medicine Center, the Third Xiangya Hospital, Central South University, Changsha, Hunan China; 3 Reproductive Medicine Center, Department of Obstetrics and Gynecology, the Second Xiangya Hospital, Central South University, Changsha, Hunan China

**Keywords:** Molecular biology, Pathogenesis

## Abstract

Structural defects in the zona pellucida (ZP), caused by mutations in *ZP* genes, are a recognized cause of female infertility; however, their pathogenic mechanisms are not fully understood. Here, we investigated how two distinct ZP defects (complete absence and thinning) compromise fertility using *Zp1*^mut/mut^ and *Zp2*^mut/mut^ rat models. We found that ZP deficiency leads to stage-specific oocyte loss during early antral follicle development in vivo and arrests the maturation of fully grown oocytes in vitro, which also exhibit reduced diameter and mitochondrial dysfunction. From the secondary follicle stage onward, granulosa cells showed reduced proliferation, increased apoptosis, and impaired adhesion, culminating in a disorganized cumulus-oocyte complex morphology and disrupted steroidogenesis by the antral stage. Further analysis revealed that the specialized structures for oocyte-somatic cell interaction, namely transzonal projections and oocyte microvilli, were disorganized and reduced in number. This structural disruption was accompanied by a global perturbation of the bidirectional communication and physical adhesion network between the oocyte and its somatic niche, underscoring the ZP’s essential role in organizing this functional microenvironment. At the molecular level, single-cell transcriptomic and protein analyses demonstrated that ZP deficiency induces a thinning of the oocyte cortical actin layer and dysregulation of cytoskeletal dynamics. This was associated with an upregulation of actin-regulating proteins, including TPM4 and ACTN1, and the engagement of focal adhesion-related pathways. The observed cortical actin disorganization provides a plausible mechanistic link to the concurrent abnormalities in microvilli and cell-cell adhesion. Collectively, our results establish the ZP as a critical structural scaffold that ensures oocyte cortical integrity and coordinates the surrounding somatic cell niche. Its disruption leads to a progressive failure in oocyte-somatic cell interaction and support, ultimately resulting in oocyte developmental impairment and loss. This study provides detailed mechanistic insights into the pathogenesis of ZP-related female infertility (particularly empty follicle syndrome).

## Introduction

The zona pellucida (ZP), a highly conserved extracellular matrix (ECM) enveloping mammalian oocytes, zygotes, and preimplantation embryos, is synthesized and dynamically remodeled by the oocyte during folliculogenesis [1]. It is well-established for its crucial roles in mediating sperm recognition, inducing the acrosome reaction, preventing polyspermy, and protecting the developing embryo before implantation [2–4]. However, its functions during oocyte development remain poorly understood [5, 6]. Clinically, severe ZP defects, such as complete absence or thinning caused by mutations in human *ZP* genes, are linked to female infertility. While some aspects of these phenotypes align with the known fertilization-related roles of the ZP, others cannot be fully explained by these functions alone [7–13]. Furthermore, other ZP abnormalities, such as an agar-like texture, are also associated with mild oocyte quality deterioration [14, 15]. These observations collectively imply an important role for the ZP in oocyte development, yet the underlying mechanisms are still unclear.

The mouse is a suboptimal model for such investigations, as phenotypes resulting from *Zp* gene defects in mice diverge from those observed in humans [16–18]. To bridge this gap, our team employed the rat model, which possesses four *Zp* genes (*Zp1-4*) like humans, unlike mice, which have only three (*Zp1-3*) [19]. We previously generated rat models with mutations in *Zp1* [20] and *Zp2* [21]. These models exhibit ZP defects and associated infertility phenotypes that closely recapitulate the clinical observations [8, 11]. Therefore, using these established rat models, we aim to investigate how the severe ZP defects caused by *Zp1* or *Zp2* mutations impact oocyte development and to elucidate the underlying molecular mechanisms.

Under physiological conditions, the ZP begins to form at the primary follicle stage and gradually thickens thereafter [1, 6]. Its deficiency may therefore affect the entire course of folliculogenesis. Studies in mice and rats with *Zp* gene abnormalities consistently show a reduction in antral follicle number, suggesting a critical impact at this stage [17, 21]. Several key questions, however, remain unanswered: Does ZP deficiency specifically impair a particular substage of the antral phase or affect it broadly? Is the quality of the few oocytes that reach the antral stage compromised, and can they achieve normal maturation through in vitro culture? Additionally, the mechanism underlying the high incidence of empty follicle syndrome (EFS) in patients with *ZP* mutations requires clarification [22]. The ZP forms a physical microenvironment that alters the spatial relationship between the oocyte and granulosa cells (GCs). It provides a scaffold for specialized cellular projections, such as transzonal projections (TZPs) from GCs and microvilli from the oocyte, which are essential for oocyte development [23–27]. Although we previously observed abnormal TZP structure in antral follicles of *Zp*‑mutant rats [20, 21], how severe ZP defects affect the formation and function of these projections throughout follicular development remains to be systematically elucidated. We thus hypothesize that the loss of ZP integrity impairs oocyte development not merely by a passive physical absence, but by actively disrupting the organization and function of the specialized structures (TZPs and microvilli), which in turn perturbs the bidirectional signaling and metabolic support from GCs throughout folliculogenesis [28–31].

To address these questions, we conducted a systematic, stage-by-stage analysis of oocyte and GC development, the architecture of their specialized structures (microvilli and TZPs), intercellular communication, as well as the underlying molecular mechanisms via single-cell transcriptomic sequencing. Our results demonstrate that the ZP is critical for maintaining the oocyte cortical actin cytoskeleton and organizing a functional bidirectional communication microenvironment. Disruption of this axis provides a mechanistic explanation for the oocyte defects and infertility associated with ZP abnormalities.

## Results

### Follicular development and oocyte meiotic competence in ZP-deficient rats

**Fig. 1:**
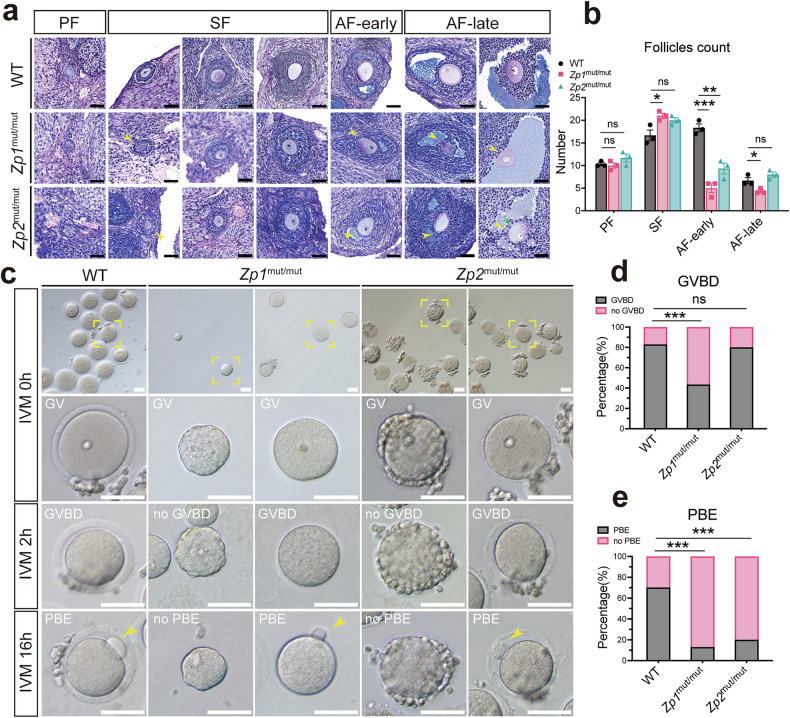
*Zp1*^mut/mut^ and *Zp2*^mut/mut^ rats display defects in folliculogenesis and fail to complete oocyte meiotic maturation. **a** Representative PAS-stained images of follicular development at different stages (triangular yellow arrow: abnormally organized GCs around the oocyte; green one-way arrow: the ectopic GC). **b** Follicle counts at different stages in ovarian sections from WT, *Zp1*^mut/mut^, and *Zp2*^mut/mut^ rats; *n* = 3 rats (10 sections per rat) per genotype. **c** Representative images of oocytes from WT, *Zp1*^mut/mut^, and *Zp2*^mut/mut^ rats during in vitro maturation, cultured 0 h to 16 h (triangular yellow arrow: first polar body). **d**, **e** Rates of germinal vesicle breakdown (GVBD) and polar body extrusion (PBE) rates of GV oocytes; *n* = 158, 23, and 60 oocytes (from 6 rats per genotype) for WT, *Zp1*^mut/mut^, and *Zp2*^mut/mut^ groups, respectively. Data are expressed as mean ± SEM. *P < 0.05; **P < 0.01; ***P < 0.001; ns none significant. Scale bar, 50 µm. PF primary follicle, SF secondary follicle, AF antral follicle.

We confirmed that histological analysis had revealed a significant reduction in antral follicle numbers in both *Zp1*^mut/mut^ and *Zp2*^mut/mut^ rats compared to wild-type (WT) controls (Fig. S1e). In this study, this reduction was associated with a decreased yield of fully grown germinal vesicle (GV) stage oocytes (Fig. S1b). When antral follicles were stratified into early and late stages, both mutant types exhibited a significant reduction in early antral follicle counts (Fig. 1a, b). Notably, the number of late antral follicles remained unaffected in *Zp2*^mut/mut^ rats. The oocyte diameter increase from early to late antral stages did not differ significantly among genotypes (Fig. S1g). To assess meiotic maturation, GV oocytes were subjected to in vitro maturation (IVM) (Fig. 1c). *Zp1*^mut/mut^ oocytes displayed a markedly reduced germinal vesicle breakdown (GVBD) rate, while both *Zp1*^mut/mut^ and *Zp2*^mut/mut^ oocytes had significantly lower polar body extrusion (PBE) rates than WT (Fig. 1d, e). Morphological inspection of oocytes revealed that *Zp1*^mut/mut^ oocytes frequently lacked GC encapsulation, exhibited increased fragility, and occasionally showed cytoplasmic leakage (Fig. 1a, c, Fig. S1a). In contrast, some *Zp2*^mut/mut^ oocytes exhibited excessively tight GC adhesion or were found protruding from a thinned or ruptured ZP. Additionally, fully grown GV oocytes from both mutants were significantly smaller than those from WT, with *Zp1*^mut/mut^ oocytes showing the most pronounced size reduction (Fig. S1c).

### ZP deficiency is associated with mitochondrial dysfunction and a pro-apoptotic shift in oocytes

**Fig. 2:**
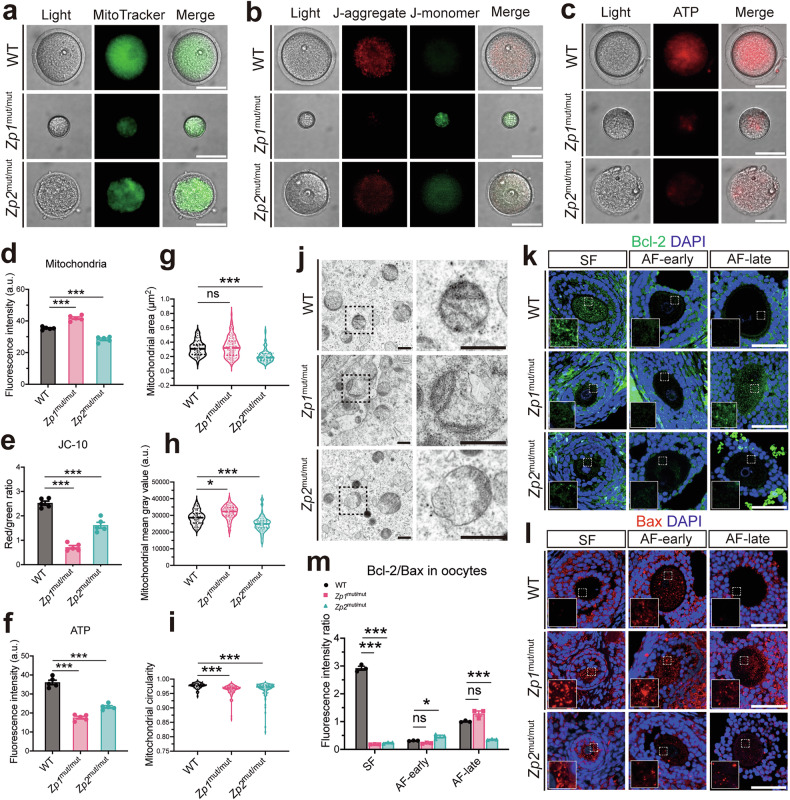
ZP deficiency is associated with mitochondrial alterations and a pro-apoptotic shift in oocytes. **a** Representative images of mitochondrial distribution in GV oocytes from WT, *Zp1*^mut/mut^, and *Zp2*^mut/mut^ rats. **b** Assessment of mitochondrial membrane potential in GV oocytes by JC-10 staining (red: active mitochondria; green: inactive mitochondria). Representative images are shown. **c** Representative images of ATP distribution in GV oocytes. Quantitative analyses of fluorescence intensity in GV oocytes: **d** total mitochondrial signal, **e** ratio of red/green JC-10 fluorescence (membrane potential), and **f** ATP signal; *n* = 5 oocytes (pooled from ≥3 rats) per genotype. Morphometric analysis of mitochondria from TEM: **g** area, **h** mean gray value, and **i** circularity; Mitochondrial counts: 70, 126, and 108 (from one rat per genotype, respectively). **j** Representative TEM images of mitochondria in oocytes. Representative immunofluorescence images of Bcl-2 (**k**) and Bax (**l**) in growing oocytes from follicles at different stages. **m** Fluorescence intensity ratio of Bcl-2 to Bax in growing oocytes; *n* = 3 follicles (from 3 rats) per genotype. For (**d**–**f** and **m**), data are expressed as mean ± SEM; for (**g**–**i**), data are presented as median with 25th and 75th percentiles. *P < 0.05; **P < 0.01; ***P < 0.001; ns none significant. Scale bars: 50 μm (**a**–**c**, **k**, **l**); 0.5 μm (**j**).

Given the established role of mitochondria in oocyte development and maturation [32], we isolated GV oocytes to assess mitochondrial function (Fig. 2a–j). GV oocytes from both *Zp1*^mut/mut^ and *Zp2*^mut/mut^ rats showed dense, unevenly distributed aggregates of MitoTracker Green-labeled mitochondria, contrasting with the homogeneous pattern in WT oocytes (Fig. 2a). Signal intensity was decreased in *Zp2*^mut/mut^ oocytes but increased in *Zp1*^mut/mut^ compared with WT (Fig. 2d). Mitochondrial membrane potential, measured by JC-10 staining, was reduced in *Zp1*^mut/mut^ and *Zp2*^mut/mut^ oocytes compared with WT (Fig. 2b, e), along with a decrease in ATP levels (Fig. 2c, f). Transmission electron microscopy (TEM) revealed mitochondrial swelling and rupture in oocytes of *Zp1*^mut/mut^ and *Zp2*^mut/mut^ rats (Fig. 2j). Quantitative analysis of mitochondrial morphology further showed that mitochondrial area was unchanged in *Zp1*^mut/mut^ but decreased in *Zp2*^mut/mut^ oocytes (Fig. 2g). The mean gray value (reflecting matrix density) increased in *Zp1*^mut/mut^ but decreased in *Zp2*^mut/mut^ mitochondria (Fig. 2h). Moreover, mitochondrial circularity was reduced in both mutants, indicating a departure from the normal rounded shape (Fig. 2i). Together, these data indicate that distinct pathological changes underlie the disruption of mitochondrial architecture and function in ZP-deficient oocytes.

Given the link between mitochondrial dysfunction and apoptosis, and our previous observation of increased apoptosis in ovulated oocytes of *Zp2*^mut/mut^ rats [21], we next investigated whether this abnormality originates during folliculogenesis via the mitochondrial pathway. We assessed apoptosis execution (by TUNEL assay) and the expression of key apoptotic regulators, Bcl-2 (anti-apoptotic) and Bax (pro-apoptotic), in oocytes at this earlier developmental stage. Immunofluorescence revealed a significantly decreased Bcl-2/Bax ratio in oocytes of both mutants at the secondary follicular stage (Fig. 2k–m). Importantly, this pro-apoptotic shift was not accompanied by detectable TUNEL signals in the oocytes themselves (Fig. S1i). These results collectively suggest that the activation of the mitochondrial apoptotic pathway in mutant oocytes represents an early cellular stress response, which is associated with subsequent developmental compromise rather than immediate apoptotic execution. Intriguingly, *Zp2*^mut/mut^ oocytes exhibited a transient increase in the Bcl-2/Bax ratio at the early antral stage, followed by a decrease later. In contrast, the ratio in *Zp1*^mut/mut^ oocytes showed no significant changes across these stages. This differential pattern suggests that the completely absent compared to the thinned ZP may distinctly modulate oocyte survival signaling over time. Consistent with the progressive nature of this stress response, TUNEL-positive oocyte nuclei were occasionally observed in late antral follicles of both genotypes (Fig. S1i). However, due to the limited number of oocytes available for analysis at this advanced stage (*n* = 3 per group), a quantitative comparison of the incidence between genotypes was not feasible.

### ZP defects induce both structural disorganization and functional deficits in GCs

**Fig. 3:**
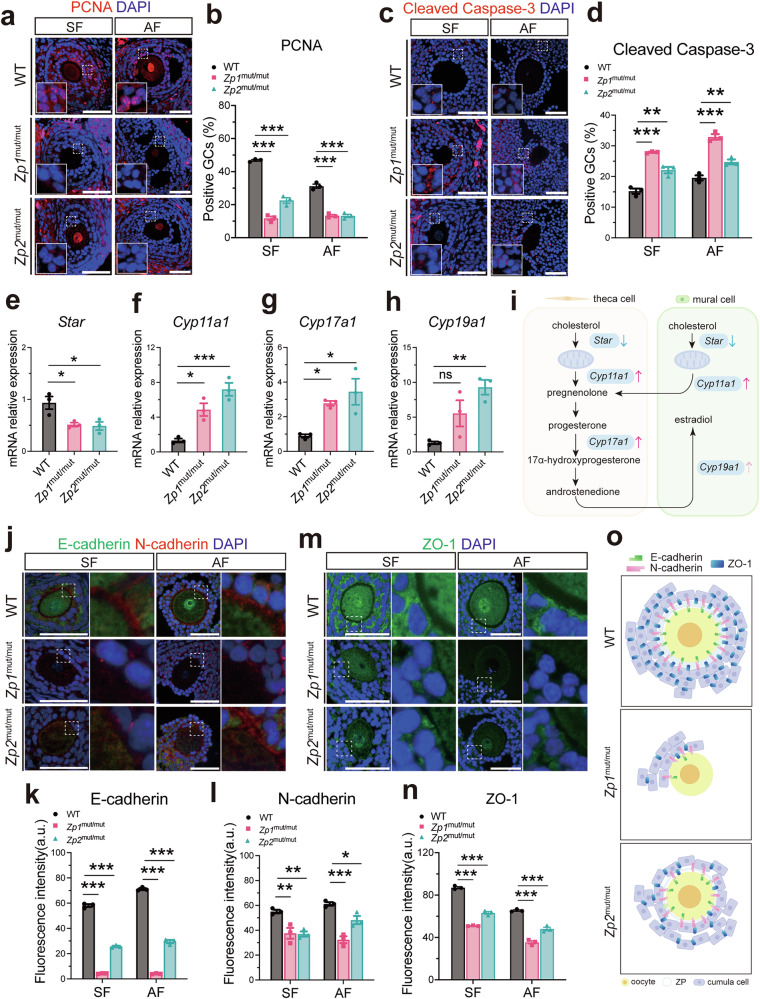
ZP deficiency correlates with functional impairment and structural disorganization in GCs. **a** Representative immunofluorescence images of PCNA in GCs from follicles at different developmental stages. **b** Quantification of GC proliferation by PCNA-positive cell rate. **c** Representative immunofluorescence images of Cleaved Caspase-3 in GCs from follicles at different stages. **d** Quantification of GC apoptosis by Cleaved Caspase-3-positive cell rate. **e**–**h** mRNA expression levels of steroidogenic genes (*Star, Cyp11a1, Cyp17a1, Cyp19a1*) in ovaries. **i** Schematic illustration of the altered expression of steroidogenic genes in somatic cells of mutant rats. **j** Representative immunofluorescence images of E-cadherin and N-cadherin in follicles at different stages. Fluorescence intensity of **k** E-cadherin in oocytes and **l** N-cadherin in GCs. **m** Representative immunofluorescence images of ZO-1 in GCs from follicles at different stages. **n** Quantification of ZO-1 fluorescence intensity in GCs. **o** Schematic illustrating the altered structural organization of GCs around the oocyte in mutant cumulus-oocyte complexes (COCs). **a**–**d**, **j**–**n**: *n* = 3 follicles (from 3 rats) per genotype; **e**–**h**: *n* = 3 rats per genotype. Data are expressed as mean ± SEM. *P < 0.05; **P < 0.01; ***P < 0.001; ns none significant. Scale bar, 50 µm.

Given the vital role of GCs in follicular development, we then assessed their proliferation, apoptosis, and steroidogenic capacity (Fig. 3). In both *Zp1*^mut/mut^ and *Zp2*^mut/mut^ rats, immunofluorescence at the secondary and antral follicular stages revealed a significant downregulation of the proliferation marker PCNA and a concomitant upregulation of the apoptosis execution marker Cleaved Caspase-3 in GCs (Fig. 3a–d), indicating impaired proliferation and enhanced apoptosis. Consistent with this pro-apoptotic shift, we observed a trend toward increased TUNEL-positive GCs in mutant follicles, though this difference did not reach statistical significance, likely due to the limited sample size (*n* = 3 follicles per group, Fig. S1i, j). During the antral follicle stage, GCs differentiate into cumulus cells and mural cells. Cumulus cells, together with the oocyte, form the cumulus-oocyte complex (COC), while mural cells collaborate with theca cells to secrete steroid hormones, further regulating follicular development [33, 34]. We measured the mRNA expression levels of key steroidogenic enzymes, including *Star*, *Cyp11a1*, *Cyp17a1*, and *Cyp19a1*, by quantitative real-time PCR (qRT-PCR) (Fig. 3e–i). In *Zp1*^mut/mut^ and *Zp2*^mut/mut^ rat ovaries, *Star* mRNA levels were downregulated, whereas *Cyp11a1*, *Cyp17a1*, and *Cyp19a1* exhibited dysregulated expression with varying degrees of upregulation. This pattern indicates a dysregulated steroidogenic program in mutant ovaries.

Histomorphological analysis revealed that with the progressive expansion of the GC layers during follicular development, abnormal GC arrangement became evident around oocytes with ZP defects (Fig. 1a). These abnormalities were most pronounced at the antral follicle stage. In oocytes lacking a ZP, cumulus cells frequently failed to encapsulate the oocyte. In contrast, oocytes with a thinned ZP were surrounded by disorganized and loosely arranged cumulus cell layers. Notably, in some instances, cumulus cells were mislocalized, appearing within or on the inner side of the ZP matrix instead of being confined to the outer layer. To investigate the molecular basis underlying the disorganized GC layer, we examined the expression of key intercellular junction proteins, including the adherens junction components E-cadherin and N-cadherin, and the tight junction marker ZO-1 [35]. In mutants, immunofluorescence showed a marked reduction of E-cadherin in oocytes, accompanied by decreased expression of N-cadherin and ZO-1 in the GCs (Fig. 3j–o). These findings suggest that ZP abnormalities compromise the molecular architecture of intercellular junctions, which may underlie the disrupted integrity and organization of the GC layer.

### ZP defects lead to disrupted oocyte-somatic communication and abnormal microvilli/TZPs

**Fig. 4:**
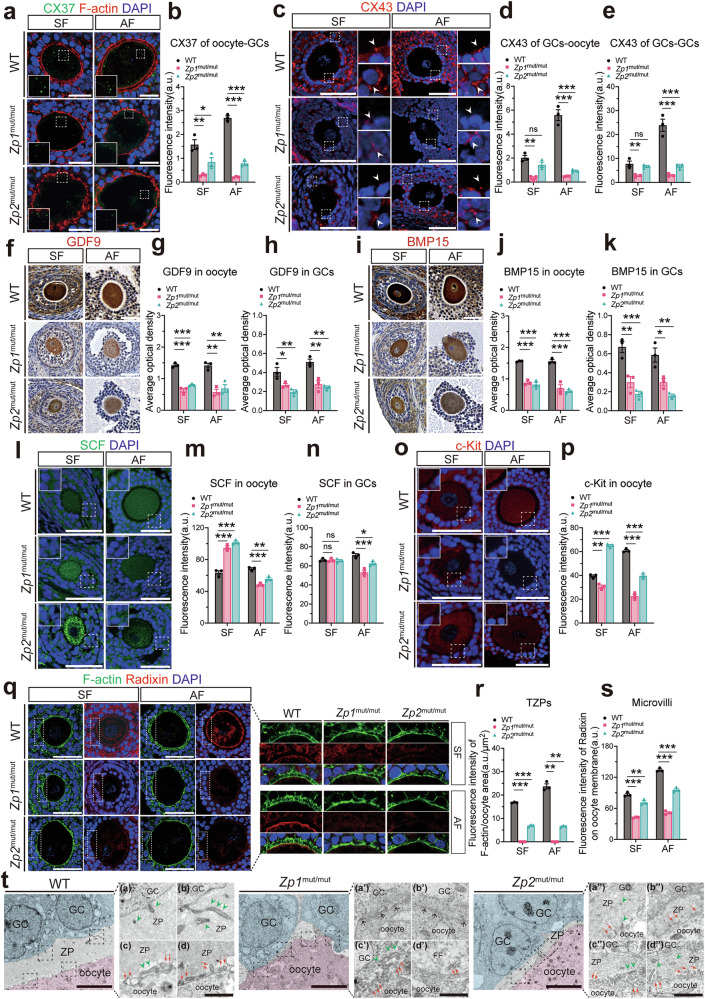
ZP deficiency is linked to dysregulated oocyte-GC communication and aberrant ultrastructural projections during folliculogenesis. **a** Representative immunofluorescence images of CX37 in oocytes from follicles at different stages. **b** Quantification of CX37 fluorescence intensity in oocytes. **c** Representative immunofluorescence images of CX43 in follicles at different stages. Quantification of CX43 fluorescence intensity in **d** GC-oocyte and **e** GC-GC gap junctions. **f** Representative immunohistochemistry images of GDF9 in follicles at different stages. Average optical density of GDF9 in **g** oocytes and **h** GCs. **i** Representative immunohistochemistry images of BMP15 in follicles at different stages. Average optical density of BMP15 in **j** oocytes and **k** GCs. **l** Representative immunofluorescence images of SCF in follicles at different stages. Quantification of SCF fluorescence intensity in **m** oocytes and **n** GCs. **o** Representative immunofluorescence images of c-Kit in oocytes from follicles at different stages. **p** Quantification of c-Kit fluorescence intensity in oocytes. **q** Co-staining of F-actin (phalloidin, marking GC-derived TZPs) and Radixin (marking oocyte-derived microvilli) in follicles at different stages. Quantification of fluorescence intensity for **r** TZPs and **s** microvilli; *n* = 3 follicles (from 3 rats) per genotype. **t** Representative TEM of the oocyte-GC interface in antral follicles. **a**, **b** The triangular green arrows indicate the cross-section of the GC-derived TZPs within the normal ZP area of the WT antral follicle; **c**, **d** The red arrows indicate the cross-section of the oocyte-derived microvilli within the normal ZP area of the WT antral follicle; **a’**, **b’** The black one-way arrows indicate gap junctions between the oocyte without ZP and GCs of the *Zp1*^mut/mut^ antral follicle; **c’**, **d’** A few abnormal forms of TZPs (triangular green arrows) and microvilli (red arrows) were present where there was a gap between the oocyte and the GC or where there was no GC coverage in the *Zp1*^mut/mut^ antral follicle (FF: follicular fluid); (**a”**–**d”**) TZPs (triangular green arrows) and microvilli (red arrows) within the thinned ZP were unevenly distributed and morphologically abnormal. Data are expressed as mean ± SEM. *P < 0.05; **P < 0.01; ***P < 0.001; ns none significant. Scale bar, 50 µm (**a**, **c**, **f**, **i**, **l**, **o**, **q**); 5 µm (**t**, overview); 1 µm (**t**, insets).

To assess how ZP defects affect intercellular communication, we analyzed marker proteins involved in gap junction (CX37/CX43) and paracrine factors (GDF9, BMP15, SCF/c-Kit) during follicular development [29]. Immunofluorescence revealed downregulated expression of the oocyte-GC gap junction proteins CX37 and CX43 (Fig. 4a–e), as well as the GC-GC gap junction protein CX43, in *Zp1*^mut/mut^ rats at the secondary follicular stage and significantly reduced at the antral stage. *Zp2*^mut/mut^ rats exhibited downregulation of these connexins at the antral stage. In addition, compared with WT, the mutants showed decreased expression of GDF9 and BMP15 in oocytes at both the secondary and antral stages, accompanied by decreased paracrine secretion to GCs (Fig. 4f–k). During the antral stage, SCF expression in GCs and its paracrine secretion to oocytes were reduced in both *Zp1*^mut/mut^ and *Zp2*^mut/mut^ rats, concomitant with c-Kit receptor in oocytes downregulation (Fig. 4l–p). Strikingly, at the secondary stage, both *Zp1*^mut/mut^ and *Zp2*^mut/mut^ rats exhibited elevated SCF secretion toward oocytes. However, *Zp1*^mut/mut^ showed lower c-Kit levels, whereas *Zp2*^mut/mut^ displayed the opposite trend. Although direct functional measurements of intercellular communication were not performed, the coordinated dysregulation of these key signaling and junctional components strongly suggests a functional impairment of the essential bidirectional crosstalk between the oocyte and its surrounding GCs in *Zp*-mutant follicles.

Given the established roles of TZPs in gap junctions and microvilli in paracrine communication [25, 36], we next examined these two ultrastructural components (Fig. 4q–s). Using phalloidin to stain actin filaments (F-actin), we assessed TZPs. From the secondary follicle stage onward, *Zp1*^mut/mut^ follicles lacked TZPs, while *Zp2*^mut/mut^ follicles exhibited sparse and disorganized TZPs. To quantify this defect, we measured the total F-actin fluorescence intensity associated with TZPs, normalized it to oocyte area, and found it to be significantly reduced. In parallel, staining for the microvillar marker Radixin revealed a marked attenuation of the fluorescent signal at the oocyte membrane in both mutants compared to WT, also beginning at the secondary follicle stage. TEM of antral follicles further detailed these defects (Fig. 4t). In oocytes completely lacking the ZP, GCs incompletely surrounded the oocyte. Where GCs made direct contact with the oocyte plasma membrane, typical TZPs and microvilli were absent, and gap junctions formed directly at these sites (Fig. 4ta’, b’). In the intercellular spaces between the oocyte and GCs, occasional, malformed TZPs and microvilli were detectable (Fig. 4tc’). In areas lacking GC coverage, the oocyte was exposed to follicular fluid, where residual, disorganized microvilli were infrequently observed (Fig. 4td’). In oocytes with the thinned ZP, both TZPs and microvilli were irregularly distributed (either sparse or clustered) and exhibited shortened, thinner morphology (Fig. 4ta”–d”). Together, these data demonstrate that ZP deficiency severely compromises the integrity and organization of TZPs and microvilli, the key ultrastructural elements mediating oocyte-somatic cell communication.

### ZP defects disrupt actin dynamics and cortical architecture in oocytes, associated with impaired microvilli formation

**Fig. 5:**
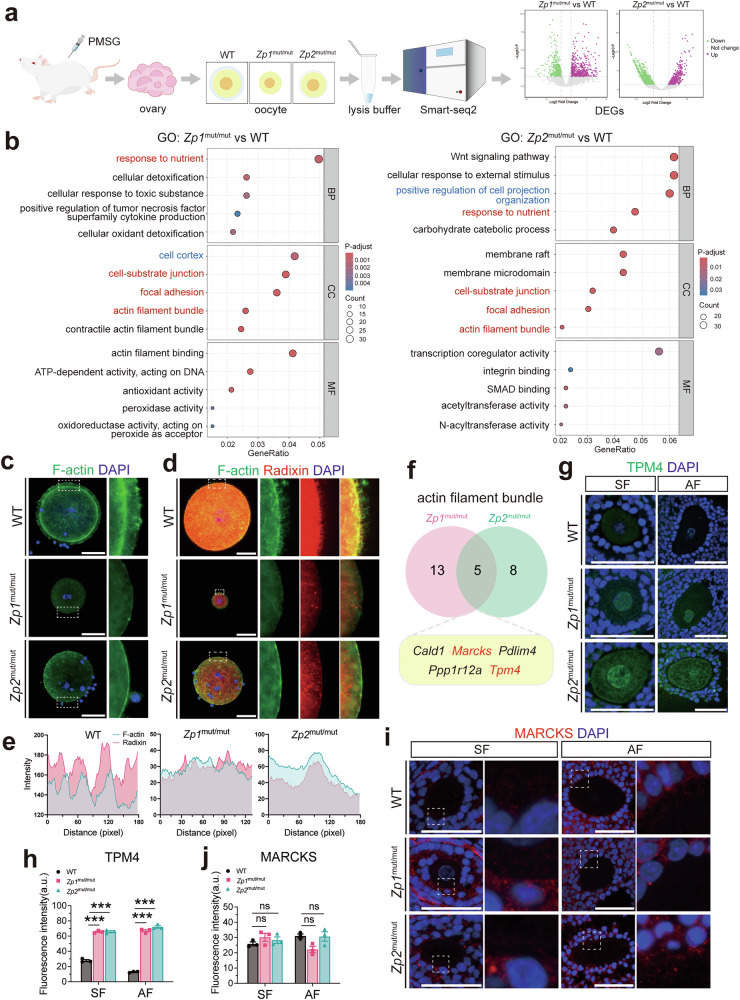
ZP defects disrupt actin dynamics and cortical architecture, associated with impaired microvilli formation. **a** Schematic of the Smart-seq2 single-oocyte transcriptomic profiling workflow. **b** GO enrichment analysis of upregulated DEGs in *Zp1*^mut/mut^ and *Zp2*^mut/mut^ GV oocytes compared to WT. **c** Staining of F-actin (phalloidin) to assess the actin cortex in GV oocytes. Analysis of F-actin and Radixin co-localization at the oocyte cortex by **d** representative co-staining and **e** line-scan fluorescence intensity profiles. **f** Venn diagram of DEGs associated with the actin filament bundle between *Zp1*^mut/mut^ and *Zp2*^mut/mut^ oocytes. **g** Representative immunofluorescence images of TPM4 in oocytes from follicles at different stages. **h** Quantification of TPM4 fluorescence intensity in oocytes. **i** Representative immunofluorescence images of MARCKS in oocytes from follicles at different stages. **j** Quantification of MARCKS fluorescence intensity in oocytes. **g**–**j** *n* = 3 follicles (from 3 rats) per genotype. Data are expressed as mean ± SEM. *P < 0.05; **P < 0.01; ***P < 0.001; ns none significant. Scale bar, 50 µm.

To gain further insight into the molecular mechanisms underlying the impaired developmental competence of oocytes with ZP defects, we performed single-cell RNA sequencing (Smart-seq2) on fully grown GV oocytes. Compared with WT, *Zp1*^mut/mut^ and *Zp2*^mut/mut^ oocytes exhibited 755 and 714 upregulated genes and 652 and 1179 downregulated genes, respectively (Fig. 5a, Supplementary Table 1). Gene Ontology (GO) analysis of upregulated differentially expressed genes (DEGs) revealed shared enrichment in the biological process ‘response to nutrient’ and the cellular components ‘cell-substrate junction’, ‘focal adhesion’, and ‘actin filament bundle’ in both mutant genotypes (Fig. 5b). Additionally, *Zp1*^mut/mut^ oocytes showed specific enrichment in the cellular component ‘cell cortex’, while *Zp2*^mut/mut^ oocytes exhibited enrichment in the biological process ‘positive regulation of cell projection organization’. These findings pointed to a potential dysregulation of the actin cytoskeleton in ZP-deficient oocytes.

To test this hypothesis, we next sought to directly quantify the integrity of the oocyte’s intrinsic cortical actin cytoskeleton (Fig. 5c). For this purpose, we analyzed isolated, fully grown GV oocytes following mechanical removal of cumulus cells. Phalloidin staining in this preparation revealed two distinct F‑actin structures: (1) the cortical actin ring beneath the oolemma, and (2) residual TZPs that remained embedded in or attached to the ZP. A profound defect was observed in the cortical actin ring. Specifically, compared to the thick, continuous ring of WT oocytes, the cortical F-actin signal was markedly thinner in *Zp2*^mut/mut^ oocytes and further diminished in *Zp1*^mut/mut^ oocytes (Fig. 5c, Fig. S1m). This thinning phenotype was especially discernible in mutant oocytes owing to a concomitant decrease in residual TZPs. In WT oocytes, the robust cortical ring was often partially interwoven with or obscured by a dense meshwork of residual TZPs, complicating its independent visualization. By contrast, and consistent with our earlier observations in tissue sections (Fig. 4q), *Zp1*^mut/mut^ oocytes displayed virtually no residual TZPs, while *Zp2*^mut/mut^ oocytes showed only sparse remnants. Together, these data provide direct evidence that ZP deficiency is associated with a cell-autonomous impairment of the oocyte’s cortical actin cytoskeleton and a concomitant loss of TZPs, supporting a model in which the ZP acts as an essential scaffold for both structures.

Our observations that ZP deficiency disrupts both the cortical actin cytoskeleton (Fig. 5c) and microvilli (Fig. 4q) led us to examine their spatial relationship. We performed co-localization staining for F-actin (phalloidin) and the microvillar marker Radixin on isolated GV oocytes (Fig. 5d, e). In WT oocytes, F-actin and Radixin signals showed a high degree of overlap at the cortex, particularly in microvilli-rich areas, with synchronized signal intensity. In both *Zp1*^mut/mut^ and *Zp2*^mut/mut^ oocytes, the significant reduction in cortical F-actin signal was accompanied by a corresponding decrease in Radixin signal. This strong correlation indicates that the loss of cortical actin integrity directly impairs Radixin localization, explaining the concurrent microvillar defects observed in ZP-deficient oocytes.

To explore potential regulators of the perturbed actin cortex, we focused on common DEGs within the ‘actin filament bundle’ category (Fig. 5f–j). Five genes were shared between mutants, including *Tpm4* (tropomyosin 4) and *Marcks*, both implicated in actin dynamics and cortical stability [37, 38]. Immunofluorescence analysis revealed a persistent upregulation of TPM4 protein in mutant oocytes from the secondary follicle stage onward. In both *Zp1*^mut/mut^ and *Zp2*^mut/mut^ oocytes, TPM4 was distributed throughout the ooplasm with prominent accumulation within the nucleus. Notably, in *Zp2*^mut/mut^ oocytes, TPM4 also exhibited discernible enrichment at or adjacent to the oocyte plasma membrane, a pattern not observed in WT or *Zp1*^mut/mut^ oocytes. In contrast, despite the upregulation of *Marcks* mRNA, quantitative immunofluorescence analysis revealed no statistically significant changes in MARCKS protein levels across genotypes at any follicular stage. These results indicate dysregulation of actin-related pathways and reveal a distinct, ZP defect-dependent alteration in TPM4 subcellular distribution.

### Upregulation of ITGA1 with coordinated membrane recruitment of Src and PFN1 in oocytes with thinned ZP

**Fig. 6:**
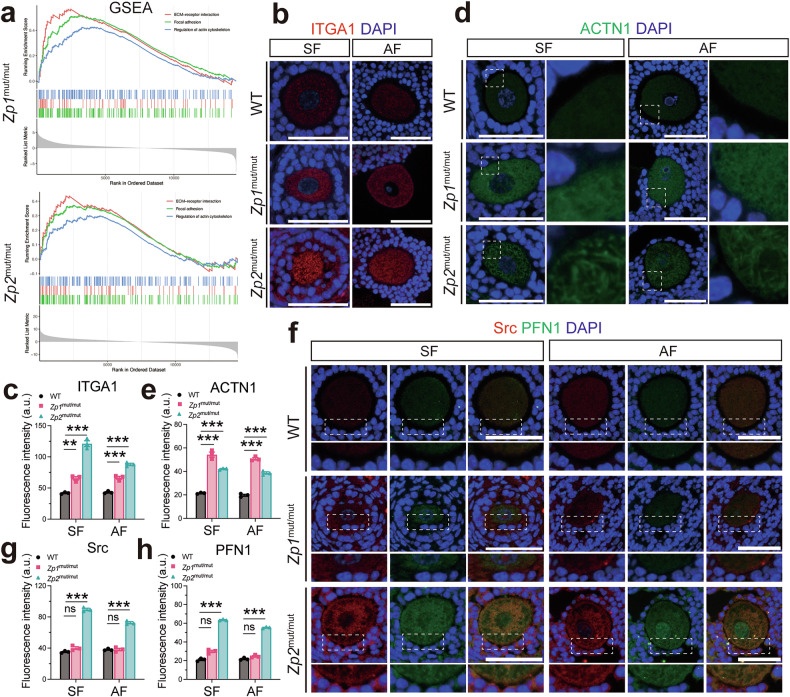
GSEA-predicted pathway alterations and validation of upregulated adhesion-cytoskeleton regulators in ZP-deficient oocytes. **a** GSEA of pathways related to ECM-receptor interaction (red), focal adhesion (green), and regulation of actin cytoskeleton (blue) in *Zp1*^mut/mut^ and *Zp2*^mut/mut^ oocytes. Expression of the ECM-receptor ITGA1: **b** representative immunofluorescence images and **c** quantification of fluorescence intensity in oocytes from follicles at different stages. Expression of the focal adhesion protein ACTN1: **d** representative immunofluorescence images and **e** quantification of fluorescence intensity. Expression and co-localization of the actin cytoskeleton regulators Src and PFN1: **f** representative co-immunofluorescence images, with quantification of **g** Src and **h** PFN1 fluorescence intensity. **b**–**h** *n* = 3 follicles (from 3 rats) per genotype. Data are expressed as mean ± SEM. *P < 0.05; **P < 0.01; ***P < 0.001; ns none significant. Scale bar, 50 µm.

To elucidate molecular mechanisms underlying the distinct cortical actin phenotypes in oocytes completely lacking versus those with a thinned ZP, we performed gene set enrichment analysis (GSEA) on our single-cell transcriptomic data (Fig. 6a). The analysis revealed significant enrichment of pathways including ‘ECM-receptor interaction’, ‘focal adhesion’, and ‘regulation of actin cytoskeleton’ in both mutant oocytes. This suggested a potential dysregulation of integrin-mediated signaling, which transduces ECM cues to remodel the actin cytoskeleton via focal adhesions [39, 40]. Based on these findings and their established roles, we selected three key regulators of this pathway for validation: the ECM-receptor integrin-alpha1 (ITGA1), the focal adhesion kinase Src, and the actin-binding protein profilin 1 (PFN1) [41–43]. Immunofluorescence confirmed upregulation of ITGA1 in oocytes of both mutant genotypes (Fig. 6b, c). We also examined α-actinin-1 (ACTN1) (Fig. 6d, e), a regulator of actin cytoskeletal stability [43, 44]. ACTN1 was significantly upregulated in the cytoplasm of both mutant oocytes, but its subcellular distribution differed: it displayed a diffuse pattern in *Zp1*^mut/mut^ oocytes but formed an organized filamentous meshwork in *Zp2*^mut/mut^ oocytes. In contrast, upregulated signals of Src and PFN1, which showed co-localization at the oocyte membrane, were specific to *Zp2*^mut/mut^ oocytes (Fig. 6f–h). Together, these data indicate that a thinned ZP permits a distinct cellular response, characterized by membrane-localized Src/PFN1 and organized ACTN1, which may underlie the relatively preserved cortical actin architecture in *Zp2*^mut/mut^ oocytes.

## Discussion

Gap junctional communication and paracrine signaling are two fundamental pillars of bidirectional communication between the oocyte and GCs, orchestrating follicular development [30, 31]. Gap junctions, assembled from connexin proteins (e.g., CX37 and CX43), provide direct channels for the exchange of small metabolites and ions [45]. After primary follicle formation, the ZP is deposited, gradually establishing a physical barrier between the two cell types [1]. Concurrently, GC-derived TZPs begin to form [23]. Together with microvilli on the oocyte surface, these structures constitute key conduits for material exchange and signaling across the ZP [27]. Within this confined space, paracrine signaling molecules, such as GDF9 and BMP15, establish dynamic local concentration gradients, forming an intricate intercellular signaling network [46]. In this study, severe ZP deficiency neither recapitulated the complete follicular developmental arrest (i.e., failure to reach the antral stage) caused by deficiency of key communication molecules such as CX37, CX43, or GDF9 [47–49], nor caused the complete absence of TZPs or microvilli, as seen in *Myo10* or *Radixin* knockout models [25, 26]. This phenotypic distinction suggests that the primary function of the ZP may not be to serve as an “absolute prerequisite” for the generation of these communication molecules or cellular projections, but rather to provide a highly organized three-dimensional spatial scaffold for these communicative processes and their associated structures.

**Fig. 7:**
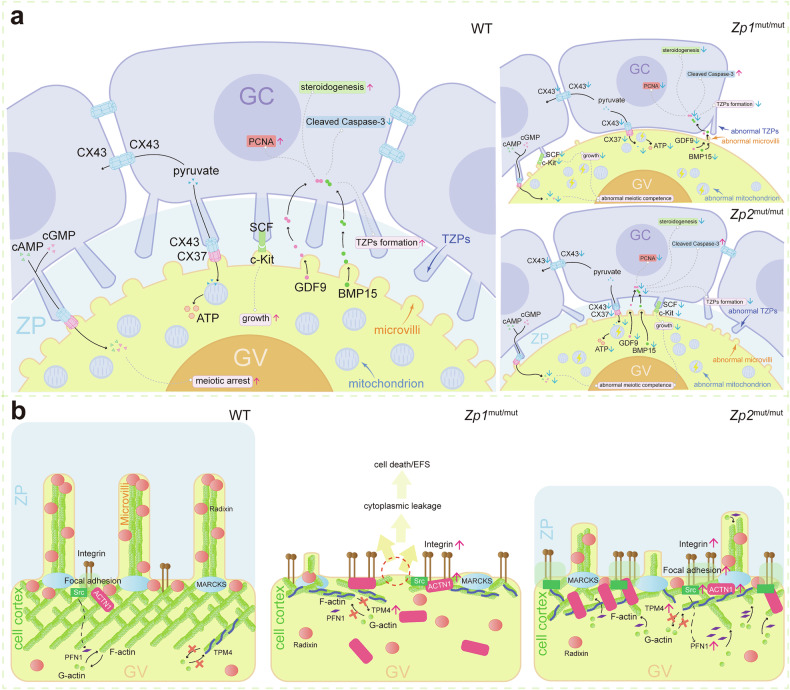
Conceptual framework linking ZP defects to oocyte dysfunction. **a** Model of communication impairment. WT (left): An intact ZP supports the organized architecture of oocyte microvilli and GC-derived TZPs, facilitating bidirectional signaling. *Zp*-mutant (right): ZP structural defects (complete absence or thinning) lead to disorganized microvilli and TZPs, compromising the oocyte-somatic communication system. **b** Model of cortical actin dysregulation and adaptive response. Primary defect: ZP deficiency results in thinning of the oocyte cortical actin layer (depicted). Associated defect: This cortical actin thinning correlates with the loss of microvilli (as linked in **a**). Cellular compensation: In response, oocytes upregulate cytoskeletal regulators. Both mutants show elevated TPM4, integrin (ITGA1), and ACTN1. Notably, *Zp2*^mut/mut^ oocytes, which retain a thinned ZP, exhibit membrane enrichment of Src and PFN1 alongside an organized ACTN1 network. This specific signature suggests a more advanced, potentially ITGA1-Src-PFN1-mediated compensatory pathway that may locally promote actin remodeling to partially stabilize the cortex. (Note that this is a schematic of the pattern, and in fact, the growing end of F-actin should be facing the plasma membrane).

While previous studies have linked TZPs to gap junction formation between the oocyte and GCs, a significant proportion of TZP termini terminate within the ZP matrix, their function remaining elusive [24]. The observation that knockout of the key TZP formation gene (*Myo10*) permits oocyte growth but disrupts maturation supports the notion that TZPs may function more to optimize and stabilize the intercellular communication interface than to initiate it [26]. Conversely, the complete absence of microvilli caused by *Radixin* knockout severely impairs follicular development and reduces oocyte yield, a phenotype that aligns with, though is less severe than, the follicular depletion observed in our ZP-deficient models [25]. Critically, emerging evidence indicates that oocyte microvilli are directly involved in the delivery of paracrine factors like GDF9, which in turn can promote TZP formation [24, 25, 50]. Therefore, ZP abnormalities likely disrupt not only the structure of individual cellular projections but also the functional reciprocity between TZPs and microvilli. This likely contributes to a global inefficiency and disorganization of bidirectional communication, thereby underscoring the essential role of the ZP as a coordinating scaffold that spatially and functionally integrates multiple communication modalities at the oocyte-somatic cell interface (Fig. 7a). This mechanism reflects the ECM’s fundamental ability to guide cell polarity. This polarity likely explains why specialized structures such as TZPs are almost exclusively found in GCs immediately adjacent to the oocyte and are rarely present in the outer cell layers [24, 51].

Our results demonstrate that the impairment of intercellular communication and structural anomalies caused by ZP deficiency emerge as early as the secondary follicle stage and are exacerbated during antral folliculogenesis, likely constituting a major cause of antral follicle loss, particularly at the early antral stage. Critically, the global communication decline coincides with the antral stage’s heightened metabolic demands, failing to sustain the oocyte’s rapid growth [52, 53]. This insufficiency may lead to selective follicle loss, diminished oocyte quality, and associated mitochondrial dysfunction, representing a potential pathological basis for related female infertility. The recovery of late antral follicle numbers in *Zp2*^mut/mut^ rats is intriguing but mechanistically unclear. Crucially, surviving oocytes still display profound cytoskeletal and functional defects, underscoring that ZP integrity is essential for oocyte quality beyond follicle survival. The formation and maintenance of an intact COC is a hallmark of antral follicle development, and its three-dimensional architecture is widely recognized as critical for oocyte metabolic support and protection [54, 55]. Consistent with our findings of impaired oocyte-GC communication, ZP deficiency also severely compromised COC morphology. This structural defect is mechanistically linked to the downregulation of key adhesion (E-cadherin, N-cadherin) and tight junction (ZO-1) proteins, which are essential for maintaining physical cohesion and force transmission within the complex [35, 56, 57]. Taken together, our findings position the ZP as a master spatial organizer that, by ensuring the proper localization and stability of intercellular connections, is indispensable for assembling and stabilizing the somatic cell niche around the oocyte. This aligns with the fundamental role of the specialized ECM in providing the topological cues necessary for tissue morphogenesis [58, 59].

Our study demonstrates that ZP deficiency leads to cortical actin cytoskeleton thinning in oocytes. This finding highlights the conserved role of the ZP as a structural ECM component in mechanically stabilizing the oocyte cortex, aligning with the fundamental role of the pericellular matrix in providing primary structural support to resist cortical tension and maintain cytoskeletal integrity [51, 59–61]. We propose a putative two-tiered mechanistic model. First, the loss or alteration of the ZP, as a critical pericellular ECM, may directly disrupt the physical constraints and tensile forces on the oocyte membrane, leading to rapid, passive reorganization of the underlying actin network. Second, this mechanical perturbation is sensed by the cell, triggering what appear to be compensatory biochemical responses. This is supported by our single-cell transcriptomic data showing significant enrichment of genes related to ‘cell-substrate junction’ and ‘focal adhesion’, pathways central to integrin-mediated mechanotransduction [42]. Similar transcriptomic shifts were observed in human oocytes with an agar-like ZP, suggesting a conserved mechanosensitive response to ZP abnormalities [62].

Following the initial physical disruption of cortical actin architecture due to ZP deficiency, our data reveal a complex, multi-layered compensatory response aimed at restoring cytoskeletal integrity. We observed a consistent upregulation of TPM4 and ACTN1 in both mutant models. TPM family proteins are known to stabilize existing F-actin filaments and regulate their branching, while ACTN1 cross-links and bundles filaments [37, 43]. Their increased expression likely represents a cellular strategy to reinforce the remaining cortical network against thinning. However, sustained overexpression may suppress normal actin turnover, thereby impairing cytoskeletal dynamics and cortical tension [44]. Furthermore, the transcriptional upregulation of *Marcks* without a corresponding increase in protein levels exemplifies the partially uncoordinated nature of this response and may result in inadequate actin-membrane crosslinking [38]. In *Zp2*^mut/mut^ rats, however, this adaptive response appeared more advanced, potentially because the residual ZP structure may partially sustain signaling through focal adhesion pathways. This is supported by the specific co-upregulation of PFN1 (which promotes actin polymerization and facilitates the formation of cellular protrusions) and the membrane enrichment of Src, a key integrin downstream signal transducer [41–43, 63]. Concurrently, elevated levels of the integrin subunit ITGA1 in both models suggest an enhanced attempt to engage with the residual or altered ECM [42]. Given that ACTN1 can anchor integrin-based adhesion sites to the cytoskeleton, the co-upregulation of ITGA1 and ACTN1 implies a coordinated effort to strengthen the mechanical link between the cell and its pericellular matrix, potentially facilitating an ITGA1-Src-PFN1 axis to promote localized actin polymerization and cortical stabilization [41, 42, 63–65]. Therefore, the upregulation of this protein set may represent a broader, albeit poorly understood, compensatory program that the oocyte enacts to cope with ECM instability (Fig. 7b). Deciphering the hierarchy and causality within this program remains a major challenge, given the inherent complexity of cytoskeletal regulation in the specialized oocyte context and the current technical limitations. Future progress will likely depend on the development of advanced live-cell imaging and mechanical interrogation techniques tailored to this delicate system.

Critically, the persistent instability of the cortical actin cytoskeleton has important functional implications. This network serves as the essential structural foundation for both microvilli formation and the proper localization of adhesion molecules such as E-cadherin [61, 66–68]. Its disorganization thus provides a unified explanation for the co-occurrence of microvilli abnormalities and disrupted cell-cell adhesion in our models, linking upstream mechanosensing failure to a broad spectrum of oocyte-somatic cell interaction defects. Furthermore, the thinning cortical actin architecture likely compromises the oocyte’s morphological integrity (Fig. S1k, l), making the oocyte more susceptible to mechanical stress and potential rupture due to uneven force distribution [61]. This offers a plausible cellular explanation for the loss of oocytes within follicles, which aligns with the clinical presentation of EFS, where morphologically intact follicles yield no oocytes upon retrieval. The precise mechanical consequences of this actin thinning, including its effects on cortical tension and elasticity, await elucidation through future live-cell mechanical interrogation.

In summary, our findings position the ZP as a complex scaffold that not only organizes intercellular interaction but also regulates the oocyte’s cortical cytoarchitecture. These findings thereby provide new experimental evidence and a multi-layered mechanistic framework for understanding ZP-related infertility pathologies. Future studies should investigate how the biophysical properties of the ZP are translated, via specific mechanosensors, into the cytoskeletal and gene expression changes observed here, offering new perspectives for addressing fertility disorders linked to ZP dysfunction.

## Methods and materials

### Animals

The *Zp1*^mut/mut^ and *Zp2*^mut/mut^ rat models used in this study were those previously generated and characterized by our laboratory using CRISPR-Cas9-mediated genome editing [20, 21] (related human case reports refer to [8, 11]). All experiments herein were conducted using these established mutant lines and their WT littermate controls. Only sexually mature female rats (aged 8–12 weeks) were used. All rats were maintained in a temperature- and light-controlled room (23 ± 1 °C; 12:12 h light-dark cycle) with food and water provided ad libitum. Animal protocols were approved by the Animal Care and Use Committee, Central South University, China.

### Antibodies and dyes

Rabbit polyclonal Radixin antibody (ab52495) was purchased from Abcam, UK. Mouse monoclonal E-cadherin antibody (60335), PFN1 antibody (67390), TPM4 antibody (67244), and rabbit polyclonal Bcl-2 antibody (26593), Bax antibody (50599), PCNA antibody (24036), Cleaved Caspase-3 antibody (25128), N-cadherin antibody (22018), c-Kit antibody (241313), and MARCKS antibody (20661) were purchased from Proteintech, USA. Rabbit polyclonal CX43 antibody (340279) was purchased from ZENbio, China. Rabbit polyclonal CX37 antibody (ER1901-85), ITGA1 antibody (ER1911-50), and ACTN1 antibody (EM1901-52) were purchased from Huabio, China. Rabbit polyclonal GDF9 antibody (bs-1795R) and SCF antibody (bs-0545R) were purchased from Bioss, China. Rabbit polyclonal BMP15 antibody (BS70790) was purchased from Bioworld, China. Mouse monoclonal ZO-1 antibody (M900002) and rabbit polyclonal Src antibody (R013616) were purchased from Epizyme Biotech, China. JC-10 (FXP134-100) was purchased from 4A Biotech, China. MitoTracker Green (MX4309) was purchased from MaoKang Bio, China. ATP-red live cell dye (SCT045) was purchased from Sigma-Aldrich, USA. Dye phalloidin conjugates (YP0059S) were purchased from Uelandy, China. Apoptosis in follicular cells was determined using a one-step TUNEL apoptosis detection kit (CX108, Epizyme biotech, China).

### Oocyte collection and in vitro maturation

The ovaries from female rats were extracted 44 h after intraperitoneal injection of 40 IU pregnant mare serum gonadotropin (Ningbo Sansheng Pharmaceutical Corporation, China). Fully grown GV oocytes were obtained by first collecting COCs via follicle puncture, and then mechanically removing the surrounding cumulus cells through gentle pipetting with a mouth pipette. For IVM culture, the oocytes were cultured in M2 medium (Sigma-Aldrich, USA) under liquid paraffin oil (Sigma-Aldrich, USA) for 16 h at 37 °C in an atmosphere of 5% CO₂.

### Histological analysis of ovaries and follicle counting

Ovaries were collected from each group of rats and fixed overnight at 4 °C in 4% paraformaldehyde. After dehydration, tissues were embedded in paraffin for sectioning or in optimal cutting temperature compound for cryosectioning. Paraffin-embedded ovaries were sectioned at 4 μm thickness, while cryosections were cut at approximately 10 μm. From each ovary, 50 serial sections were prepared, with the largest cross-sectional area selected for analysis. Using systematic random sampling, 10 ovarian sections per group were subjected to periodic acid-Schiff (PAS) staining (Bioss, China) to perform follicular counting. Follicle classification was simplified based on established staging criteria: primary follicles (Type 3), secondary follicles (Types 4–5), early antral follicles (Type 6), and late antral follicles (Types 7–8) [69].

### Immunostaining/immunofluorescence staining

For paraffin sections, dewaxing was performed in xylene, followed by rehydration through a descending ethanol series. Endogenous peroxidase activity was quenched with 3% hydrogen peroxide (Solarbio, China) for 10 min. Antigen retrieval was carried out by heat induction in 10 mM sodium citrate buffer (pH 6.0, Solarbio, China) using a microwave oven. Frozen sections did not require these steps. The fully grown GV oocytes were fixed overnight at 4 °C in 4% paraformaldehyde, followed by permeabilization with 0.5% Triton X-100 for 10 min and five washes with PBS. Sections or oocytes were blocked with 5% BSA (ThermoFisher Scientific, USA) at 37 °C for 30 min, then incubated with primary antibodies at 4 °C overnight. After washing five times with PBS, sections were incubated with secondary antibodies (1:200) at 37 °C for 1 h. After five additional PBS washes, slides were mounted using an antifade mounting medium that contained DAPI (Beyotime, China). Oocytes were prepared as squashed slides using the same mounting medium. All samples were imaged with an inverted fluorescence microscope (Olympus, Japan). For DAB detection, after the secondary antibody step, sections were subjected to DAB chromogenic reaction, counterstained with hematoxylin for 1 min, dehydrated through graded alcohols, and mounted for whole-slide scanning. For F-actin staining, phalloidin (1:50) was mixed with the secondary antibody and applied simultaneously. Primary antibody concentrations used in this study were as follows: Radixin (1:200), E-cadherin (1:100), PFN1 (1:200), TPM4 (1:200), Bcl-2 (1:200), Bax (1:200), PCNA (1:200), Cleaved Caspase-3 (1:200), N-cadherin (1:100), MARCKS (1:200), c-Kit (1:100), CX43 (1:200), CX37 (1:50), ITGA1 (1:200), ACTN1 (1:100), GDF9 (1:200), SCF (1:100), BMP15 (1:200), ZO-1 (1:200), and Src (1:200).

### Fluorescent staining of oocytes

The fully grown GV oocytes were incubated in dye working solution (JC-10, MitoTracker Green, or ATP-red) at 37 °C for 40 min and then washed five times with PBS. The oocytes were then transferred into droplets under liquid paraffin oil in a culture dish and observed under an inverted microscope (Yongxin, China).

### RNA isolation and qRT-PCR

Ovaries were collected from rats in each group (*n* = 3) and stored at −80 °C until analysis. For total RNA extraction, 1 ml of TRIGene Reagent (GenStar, China) was added, and the tissues were homogenized on ice using a homogenizer. cDNA was synthesized using All-in-one RT Mix (GenStar, China). qRT-PCR was performed using Fast SYBR qPCR Mix (GenStar, China) and a CFX96 Real-Time PCR System (Bio-Rad, USA). Each cDNA sample was run in technical triplicate, and the mean cycle threshold (CT) value was used for subsequent calculations. The relative mRNA levels were normalized to *Gapdh* mRNA, and fold changes were quantified using the comparative 2^−ΔΔCT^ method. Primers were synthesized by Tsingke Biotechnology (China), and their sequences are listed in Supplementary Table 2.

### TEM

The ovaries were extracted, and the follicular tissues on the ovarian wall were carefully dissected under a light microscope using a 1 mL syringe. The tissues were trimmed to approximately 2 mm × 2 mm in size without disrupting the follicles. The samples were then fixed in fixative (2.5% glutaraldehyde, pH 7.0–7.5) at 4 °C. TEM was performed by Servicebio (China).

### Smart-seq2 sample preparation and data analysis

Fully grown GV oocytes were completely isolated from cumulus cells and collected in lysis buffer (Fig. 5a). Each biological replicate (*n* = 3 per genotype) contained 3 oocytes collected from 3 individual rats, pooled for sequencing. Samples were immediately frozen in liquid nitrogen and sent to BGI (China) for Smart-seq2 analysis. Read counts data were analyzed for differentially expressed genes using the DESeq2 package (R version 4.2.0) with thresholds of |log2FoldChange| ≥ 1 and adjusted *P* < 0.05. Subsequent GO and GSEA were conducted, followed by visualization of results.

### Experimental design and independent replicates

The biological independent replication unit (*n*) is defined by the sampling level: the individual rat (animal level), follicles from distinct rats (follicle level), and oocytes pooled from multiple rats (oocyte level). Sample sizes for each experiment are provided in the figure legends. These sample sizes were chosen based on field-specific standards and the effect sizes observed in preliminary studies. Where feasible, post-hoc power analyses on key outcomes (power >0.8) support the sufficiency of the sample to detect the reported effects. The intrinsic scarcity of specific biological materials (e.g., staged follicles) necessarily constrains sample sizes in this line of research. However, for analyses involving finely staged follicular subgroups, the reduced biological heterogeneity within these precisely defined categories enhances our ability to detect large effect sizes even with limited n. We report such trends with due caution, acknowledging the potential limitations in statistical power for these specific comparisons. Random allocation of animals to groups and blinding during morphological assessment were not applicable due to the genetically predetermined experimental groups and their distinct, observable phenotypes. Procedures to mitigate bias, including randomization of sample processing order and blinding during quantitative analyses, were implemented where feasible.

### Statistical analysis

Quantitative data from at least three independent experiments are presented as mean ± SEM (or SD). Statistical analyses were performed using SPSS Statistics (version 29.0) and GraphPad Prism (version 10.3.1). For comparisons among multiple groups, the appropriate test was selected after assessing normality (using the Shapiro–Wilk test) and homogeneity of variances (using Levene’s test). For normally distributed data with equal variances, one-way ANOVA was applied, followed by Dunnett’s post hoc test for comparisons of all groups against a single control group. If the assumption of equal variances was violated, Welch’s ANOVA was used, followed by the Games-Howell post hoc test. For data that violated normality or were ordinally scaled, the Kruskal–Wallis *H* test was used, followed by Dunn’s test with adjustment for multiple comparisons. For categorical data, the Pearson *χ*² test or Fisher’s exact test (for small expected frequencies) was used for overall comparison. When the overall test was significant, pairwise comparisons were conducted using *χ*² tests with the Bonferroni correction. A two-tailed *P*-value < 0.05 was considered statistically significant. Image analysis was performed using ImageJ software (version 1.54p). Fluorescence intensity was quantified as the mean gray value (integrated density divided by area) to correct for differences in oocyte or follicle size. Cortical actin thickness, TZP length, and microvilli length were quantified by averaging measurements from four orientations (top, bottom, left, and right). To assess TZP density, the integrated fluorescence intensity of phalloidin-stained F-actin within the ZP region was measured and normalized to the oocyte cross-sectional area. Mitochondrial circularity was calculated using the formula: 4π × Area/Perimeter². For GC proliferation and apoptosis assays, the positive rate was calculated as the number of positive cells divided by the total DAPI-stained nuclei per follicle section.

### Ethics approval and consent to participate

All animal procedures were approved by the Animal Care and Use Committee of the School of Basic Medical Science, Central South University, China (Approval No.2021-KT36). All methods were performed in accordance with the relevant guidelines and regulations

## Supplementary information

Supplementary legends

Figure S1

Supplementary Table 1

Supplementary Table 2

## Data Availability

Data supporting the findings of this study are available on reasonable request from the corresponding author.
