# Supplementary material for: Zona pellucida is required for oocyte actin cortex and oocyte-somatic cell interactions during oocyte growth
Source: Cell Death Discov. 2026 Apr 20;12:264. doi: 10.1038/s41420-026-03124-9 (PMC13230721; doi:10.1038/s41420-026-03124-9)
Supplement: Supplementary file 1 — Supplementary legends [file 41420_2026_3124_MOESM1_ESM.docx]

**Supplementary Figure legend**

**Fig. S1 a** Representative images of abnormal fully-grown GV oocytes and COCs from *Zp1*^mut/mut^ and *Zp2*^mut/mut^ rats. **b**-**d** Morphometric analysis of fully-grown GV oocytes: (**b**) count, (**c**) diameter, and (**d**) ZP thickness; b: n = 6 rats per genotype; c-d: n = 167 (WT), 23 (*Zp1*^mut/mut^), and 61 (*Zp2*^mut/mut^) oocytes. **e**-**h** Follicle and oocyte analysis across developmental stages: (**e**) follicle counts at different stages in ovarian sections, (**f**) follicle diameter, (**g**) oocyte diameter, and (**h**) ZP thickness of growing oocytes; e, n = 3 rats (10 sections per rat) per genotype; f-h, follicle counts (n = 3 rats per genotype): WT (PF:31, SF:40, AF-early:41, AF-late:16), *Zp1*^mut/mut^ (PF:30, SF:63, AF-early:8, AF-late:11), *Zp2*^mut/mut^ (PF:22, SF:56, AF-early:19, AF-late:18). **i**-**j** Assessment of apoptosis by TUNEL assay: (**i**) representative images at different stages (white arrows: positive GCs; double-headed arrows: positive oocytes; asterisks: cytoplasmic fragmentation; areas demarcated by white dashed circles indicate follicles); (**j**) quantification of TUNEL-positive GC rate; n = 3 follicles (from 3 rats) per genotype. **k** Representative PAS-stained images showing degenerative growing oocytes/COCs in *Zp1*^mut/mut^ follicles. **l** A corresponding TUNEL-stained follicle section illustrating a degenerative context. White arrows indicate oocytes with abnormal morphology (from irregular cytoplasm to severe fragmentation), situated amidst TUNEL-positive somatic cells. **m**-**o** Ultrastructural measurements in GV oocytes: (**m**) cortical actin thickness, (**n**) TZP length, and (**o**) microvilli length; n = 5 oocytes (pooled from ≥ 3 rats). Data are expressed as mean ± SEM or SD. **P* < 0.05; ** *P* < 0.01; *** *P* < 0.001; ns, none significant. Scale bar, 50 μm (a, i, l); 100 μm (k).

**Supplementary Table legend**

**Supplementary Table 1. Full gene expression dataset from single-oocyte RNA sequencing.**

**Supplementary Table 2. Primers sequences for qRT-PCR.**
