# Supplementary figures and images for: Zona pellucida is required for oocyte actin cortex and oocyte-somatic cell interactions during oocyte growth
Source: Cell Death Discov. 2026 Apr 20;12:264. doi: 10.1038/s41420-026-03124-9 (PMC13230721; doi:10.1038/s41420-026-03124-9)

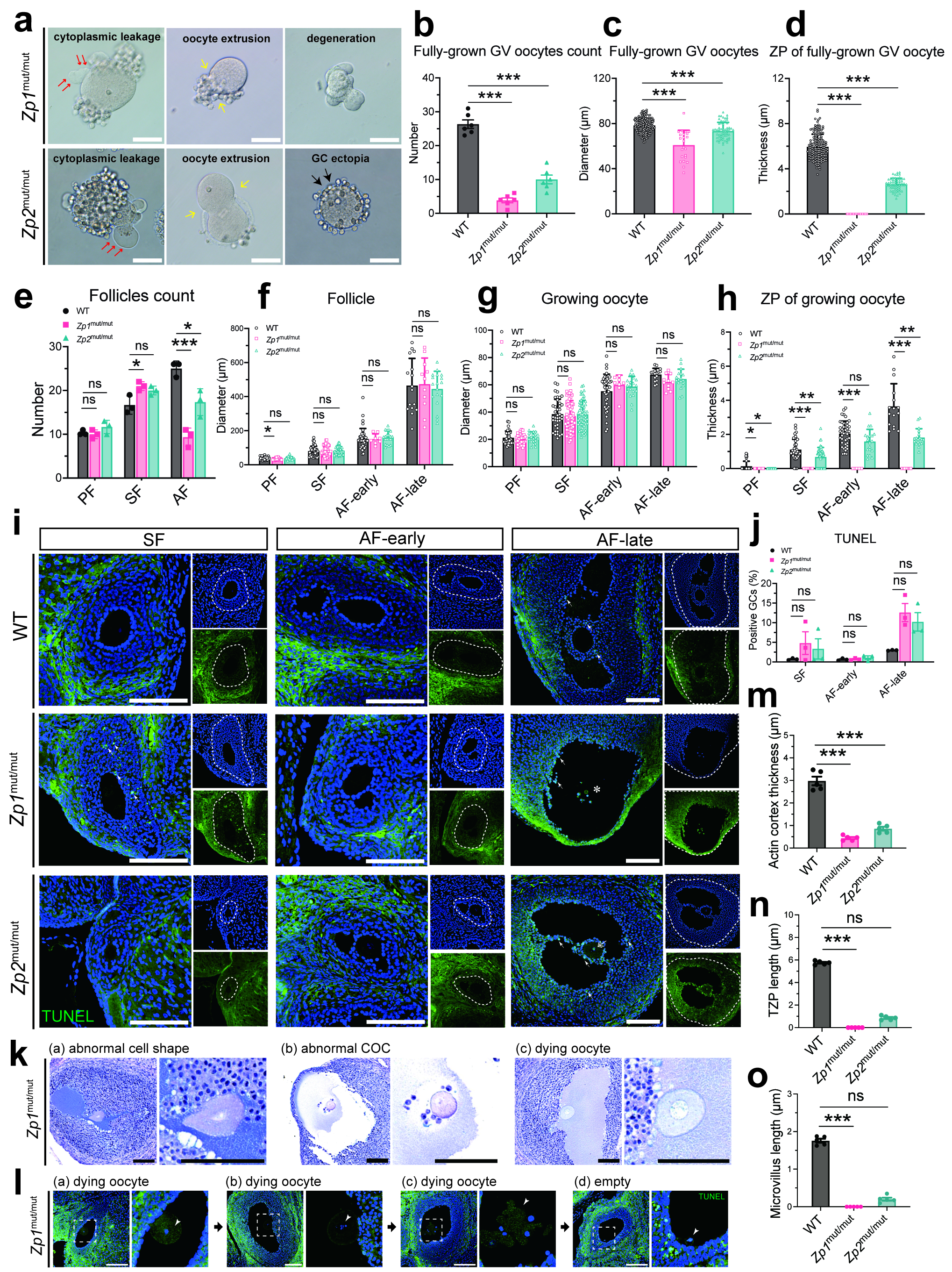

Supplement: Supplementary file 2 — Figure S1 [file 41420_2026_3124_MOESM2_ESM.tif]
